# Hormone and Microorganism Treatments in the Cultivation of Saffron (*Crocus Sativus* L.) Plants

**DOI:** 10.3390/molecules13051135

**Published:** 2008-05-13

**Authors:** Alper Aytekin, Aynur Ozkul Acikgoz

**Affiliations:** Zonguldak Karaelmas University, Bartin Forestry Faculty, 74100 – Bartin, Turkey

**Keywords:** Saffron, corm, stigma, hormone, microorganism

## Abstract

The difficult cultivation of the saffron plant (*Crocus Sativus* L.) make the spice of the same name made from its dried stigmas very valuable. It is estimated that some 75,000 blossoms or 225,000 hand-picked stigmas are required to make a single pound of saffron, which explains why it is the world’s most expensive spice. The aim of this study was to identify ways of increasing the fertility and production of saffron. For this purpose, the treatment of saffron bulbs with a synthetic growth hormone – a mixture of Polystimulins A6 and K – and two different microorganism based materials – biohumus or vermicompost and Effective Microorganisms™ (EM) – in four different ways (hormone alone, biohumus alone, EM alone and EM+biohumus) was investigated to determine whether these treatments have any statistically meaningful effects on corms and stigmas. It has been shown that EM + biohumus was the most effective choice for improved saffron cultivation.

## Introduction

The name saffron applies indistinctly to *Crocus sativus* L., a herbaceous plant with corms widespread throughout the tropical and subtropical regions of the Northern Hemisphere, and to the valuable spice obtained from the dried stigmas of this plant [1,2,3]. Saffron plants have been used for their smell, color and healing features and cultivated for their spice for over 4,000 years [4]. Currently *C. sativus* is cultivated from the Western Mediterranean to Iran, India (Kashmir), China and Japan [1]. Spain and Iran are the largest producers of the spice, accounting together for more than 80% of the world production, which is approximately 300 tons per year. In the Mediterranean region saffron is also cultivated on a much smaller scale in Italy, Greece and Turkey, where it is currently grown in the village of Davutobasi in the Safranbolu district of Karabuk province [5]. Until the first quarter of 20^th^ century the Safranbolu region was a growing and trading center for saffron, and the region is named after saffron itself [6]. 

Iran is the most productive of the Western and Central Asian cultivation areas. In recent years the productivity in this region has increased enormously and Iran now greatly outproduces Spain, to the extent that it it is widely claimed that much of the spice sold at a premium price as "Spanish saffron" is probably repackaged Iranian saffron. Nevertheless, authentic (D.O.) Spanish La Mancha Coupé saffron is still generally considered the best quality. Kashmiri saffron also has a very high reputation, but is generally unavailable outside India and furthermore, yields and quality have decreased in recent times because of the unfortunate political situation in the region. 

Saffron is used in medicine, as a food spice and in industry as a textile dye and in perfumery [7,8]. The spice has a sweetish and aromatic smell and a somewhat bitter taste [9]. The essential oil (max. 1%) contains several terpenes (pinene, cineole) and carbonyl compounds. Its most abundant constituent (50% or more) is safranal (2,6,6-trimethyl-1,3-cyclohexadiene-1-carboxaldehyde). 2-Hydroxy-4,4,6-trimethyl-2,5-cyclohexadien-1-one is another important olfactory constituent. The bitter taste is attributed to the presence of picrocrocin (4-(β-D-glucopyranosyloxy)-2,6,6-trimethyl-1-cyclohexene-1-carboxaldehyde) [2,3]. The intense color is due to cartotenoid type pigments. Although saffron contains some conventional carotenoids (α- and β-carotene, lycopene and zeaxanthin), its pronounced staining capability is mostly caused by crocetin esters; crocetin is a dicarboxylic acid with a carotenoid-like C18 backbone which is formed from carotenoid precursors (“diterpene carotenoid”). Crocin, a diester of crocin with gentobiose, is the single most important saffron pigment [10]. 

Saffron is a spice of great economic value, as one kilogram of good quality saffron produced from *Crocus sativus* L. can cost over 2,000 US dollars [11]. Approximately 150,000 flowers are needed to produce one kilogram of dried saffron, and to grow this amount one would typically need some 2,000 m^2^ under cultivation per kg harvest. The cost of Indian saffron, which has been reported to contain up to 26 times as much crocin as commercial (Sigma) saffron samples [12] costs almost 38 times less per unit dry weight compared to the latter. 

The above-ground part of the saffron plant is usually about 20-25 cm in height [13], it displays thin, long needle-shaped leaves and lasts for a year, while the bulb, which is oblate sphere in shape, surrounded by brown bark and has a diameter of 2-4 cm, lasts for many years. It germinates every year for three years forming a new plant. After the new plant blossoms and forms the following year's bulb, the above-ground part of the plant dies. Blooming starts at the 3^rd^ or 4^th^ week of October and continues until around the 15^th^ of November. 

**Figure 1:**
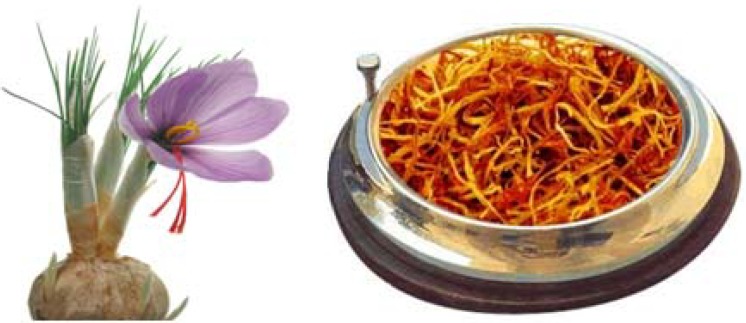
Saffron plant and its stigmas.

While some *Crocus* species can be produced from both their seeds and corms, since saffron pollen is autotriploid (2n=24), it is sterile [14], so its beautiful flowers cannot produce any seeds and propagation is possible only via corms, which makes its continued cultivation problematic. The most important part of the saffron flower (Figure 1) is its female sexual organ, composed of an ovary, ovary pipe and a three-part stigma (also called style). The stigma, deep red in color, is divided into three parts called filaments, 2.5-3.5 cm in length, having a fibrous aspect. The male part of the saffron flower, the stamens, are half the size of the stigmas, they are deep yellow and have no culinary value and are therefore much less expensive. They are frequently added as an adulterant to the red stigmas to increase the weight of commercial saffron. Legitimate powdered saffron made by grinding saffron stigmas is red-orange and under no circumstance should pure powdered saffron be any shade of yellow. 

Soil and weather conditions vary naturally in the saffron cultivating countries, as do the methods of drying the fresh saffron stigmas. Saffron production requires careful attention. Bloom collection must be performed carefully and in the early morning to facilitate the separation of the petals from the stamens and stigmas. The separated stigmas are dried at 50-80°C for 30-35 minutes [9,15]. The drying process is performed in furnaces fueled by charcoal after placing the stigmas on sheets, then the dried stigmas are cooled and collected in a dry place. The aroma, a measure of color and quality of saffron, appears during the drying process [14].

There are many comparative studies about saffron. One such study involved 270 samples of saffron from Spain and other leading producers (Greece, India, Iran, Italy and Morocco). In this study, the traditional physical parameters indicated in quality regulations were analyzed and ranges were established for each one (style length, stigma length, stigma percentage, moisture content at 40 and 103 °C, % volatile matter and % ash). It was concluded that saffron grown in the provinces of Cuenca and Albacete (Castilla-La Mancha region, Spain) showed the highest percentage of stigma length. The lower stigma mass of Spanish saffrons compared with saffron from other countries indicated that more flowers are necessary to obtain the same weight of spice. The highest values of the percentage of volatile matter were obtained in Iranian and Spanish saffrons. A greater range of ash percentage than indicated in the quality regulations was obtained [16].

As mentioned above, safranal is one of the compounds considered to be responsible for the aroma of saffron spice and measurement of its levels, has been used in the past as a measure of saffron quality although this technque is now largely discredited as a reliable measure of aroma [17,18]. In a published study [19] a "safranal value" of 269 (mostly Spanish) saffron samples was determined by means of a spectrophotometric method based on the ISO 3632 norm and the safranal content was measured using a thermal desorption-gas chromatography method. The range of variation of safranal was obtained from both methods, although there was no acceptable linear correlation between them. Among all saffron samples the Spanish ones had the highest levels and those from Iran the lowest, which might be attributed to the different processes used for dehydrating the stigmas.

The saffron spice weight produced is directly proportional to saffron plant fertility. It has been reported that the spice weight of saffron can reach 15 kg/ha in the fertilized and watered lands of Spain [20,21], while productivities of 9 kg/ha can be achieved in the Kashmir region in India, although 2-3 kg/ha production is a more typical value for the region [21]. According to a study performed in Iran, the average saffron yield is 5.4 kg/ha [22]. In another study performed in Kastamonu (Turkey) by Vurdu *et al*. [23,24] it was seen that while the number of young corms obtained from corms having a diameter of 3-5 cm was 2,070, the number of corms obtained from corms having a diameter of 1-2.9 cm was 1,579. Likewise, Cavusoglu and Erkel [25] have investigated the effect of the growing site and corm diameter on fertility and similar results have been obtained.

Modern agriculture faces the challenges of increasing growing difficulties related to a global decrease of soil fertility and increased consumer demands for ecological purity and quality products. For thess reasons there is a need for new highly effective fertilizers that preserve the ecological balance of Nature. Plant growth regulators, a product of modern biotechnology, are important in this respect. Effective Microorganisms™ (EM) is a mixed culture of beneficial microorganisms (primarily photosynthetic and lactic acid bacteria, yeast, actinomycetes and fermenting fungi) that can be applied as inoculants to increase the microbial diversity of soils. This in turn, may improve soil quality and health, which enhances the growth, yield and quality of crops. 

Biohumus is a natural organic fertilizer obtained by processing organic waste through earthworms. This fertilizer contains highly assimilated nitrogen, phosphorus, potassium, specific organic substances of humic nature, biogenic macro- and microelements and biologically active substances. Biohumus and derived products are a real alternative to mineral fertilizers and unlike these; they don’t destroy the ecological balance of the soil. Because earthworms are a natural soil component, their casings (excrement) is an inseparable part of Nature's cycle. The intensive application of mineral fertilizers, pesticides and herbicides kills earthworms and reduces their amount in soil, which in turn influences its fertility. The application of biohumus leads either to increased crop capacity and the improved crop quality or helps to improve the general state of soil. 

Plant growth regulators have been used successfully in plant breeding and between growing. While some plant growth regulators affect the interactions between physiological processes, others trigger a certain functions in plants [26]. Plant growth regulators typically increase not only plant growth, but also their resistance to environmental stresses. Polystimulins-A6 and -K (PS-A6 is auxin-like and PS-K is cytokinin-like) are synthetic compounds which show high biological activity and have many direct effects on the development and growth of plants [27,28]. Polystimulins also increase plant resistance to salt stress [29]. Demircioglu [30], Kirdar and Ertekin [31], Kirdar and Allahverdiev [32] and Demir [33] have reported that PS-A6 and PS-K treatments increased seedling growth of *Robinia pseudo-acacia* L., *Magnolia grandiflora*, *Fagus orientalis* and Black Pine seedlings, respectively. Kirdar and Ertekin also examined the growth of Atlantic cedar (*Cedrus atlantica Manetti*) grafted onto two-year old Lebanon cedar (*Cedrus libani* L.) root stocks [34]. A mixture of polystimulin (PS) growth regulator was used in small doses to determine the effects on graft success and subsequent growth during three growing seasons. The successful results showed that PS was obviously very effective. 

The aim of this study was to increase the production and the fertility of saffron. For this purpose, saffron bulbs have been treated with one hormone and two different microorganisms (polystimulin mxture, biohumus, EM and EM+biohumus) in four different ways to determine the most efficient way to obtain corms and stigmas.

## Results and Discussion

Saffron bulbs were treated with one hormone (polystimulin mixture) and two different microorganisms (biohumus and EM) in four different ways – biohumus, polystimulin and EM alone and EM+biohumus – to increase the production and the fertility of saffron. To determine the effects of the processes applied the number of corms and the dry and wet weight of stigmas have been measured. The SPSS 15.0 software package and the 95% reliability (p=0.05) One Way ANOVA test available within this software have been used to test if there was a difference between the treatments. Natural logarithms of dry and wet stigma weights have been taken to meet statistical assumptions. Thus, normal distribution and homogeneity of variances have been provided. Descriptive statistical information related to corm numbers and dry and wet stigma weights is given in Table 1.

**Table 1:** Statistical information about corm numbers, dry and wet stigma weights.

Tests	Statistical values	Types of Treatment of Saffron Corms				
		Control	Polystimulin	Biohumus	EM	EM+Biohumus
**Corm Numbers**	Avg.	2.07 **A**	2.41 **B**	2.50 **BC**	2.49 **BC**	2.59 **C**
	± s	0.952	0.948	0.941	0.978	1.023
	s^2^	0.908	0.900	0.887	0.958	1.047
	V	46.04	38.09	37.66	40.62	39.60
	N	300	300	300	300	300
**Wet Weight of Stigmas**	Avg.	2.96 **A**	3.01 **B**	3.02 **B**	3.05 **C**	3.09 **D**
	± s	0.076	0.075	0.045	0.056	0.078
	s^2^	0.006	0.006	0.002	0.003	0.006
	V	2.56	2.49	1.49	1.84	2.53
	N	300	300	300	300	300
**Dry Weight of Stigmas**	Avg.	1.49 **A**	1.61 **B**	1.59 **B**	1.77 **C**	1.86 **D**
	± s	0.201	0.119	0.245	0.203	0.236
	s^2^	0.040	0.014	0.060	0.041	0.056
	V	13.47	7.43	15.37	11.50	12.67
	N	300	300	300	300	300

Avg, average; ±s, standard deviation; s^2^, variance, V, coefficient of variation, N, number of samples used in each test; *p<0,05. Homogeneity groups: same letters in each column indicate that there is no statistical difference between the samples according to the Duncan’s multiply range test.

### 3.1. Number of Corms

To determine whether is there was a difference between the numbers of corms with or without treatment 300 bulbs were randomly from each test area. The corms for each treated group and the control sample were then counted. 

According to the One Way ANOVA (Duncan) test results it can be seen that there was a meaningful difference in the 95% reliability range between control samples and saffron treated with hormone and microorganisms. While polystimulin and EM and biohumus gave similar results it has been determined that EM, biohumus and EM+biohumus have very close results. It has been determined that EM+biohumus mixture have given the best overall result, with an average of 2.59. 

### 3.2. Weights of Wet Stigmas

Three hundred stigmas from blooms gathered from each test plot were randomly selected to determine whether there was any difference between the wet stigma weights. These were measured for each group treated with hormone and microorganisms and the control sample. The natural logarithm of the measured weight values was taken to meet the test assumptions. 

It can be seen that according to One Way ANOVA (Duncan) test results, there was a meaningful difference between control samples and saffron treated with hormone and microorganisms in the reliability range of 95%. There was also a significant difference between control samples and the polystimulin and biohumus-treated ones. There was also a meaningful difference between polystimulin and biohumus and EM. Likewise, a meaningful difference has been obtained between EM and EM+biohumus. No difference has been noted between polystimulin and biohumus. EM+biohumus gave the overall best result.

### 3.3. Weight of Dried Stigmas

Three hundred dried stigmas from blooms gathered from each test area have been randomly selected to determine whether there was a difference between the weights of dried stigmas. Weights of stigmas have been measured for each group treated with hormone and microorganism and the control set. The natural logarithms of the measured weight values were taken to meet the test assumptions. 

According to the One Way ANOVA (Duncan) test results it can be seen that there was a meaningful difference in the 95% reliability range between the control samples and saffron treated with hormone and microorganism. There was a significant difference between control samples and polystimulin and biohumus-treated ones. A meaningful difference has also been established between polystimulin and biohumus and EM. Likewise, a meaningful difference between EM and EM+biohumus has been obtained. No difference has been found between polystimulin and biohumus. EM+biohumus gave the best overall result. 

## 4. Conclusions

Since saffron is one of the most expensive spices in the world, produced in low amounts in certain regions and since saffron fertility is directly related to the spice weight of saffron, we have examined the effects of hormone and microorganism treatments on saffron corms in an attempt to increase fertility and spice production. Saffron bulbs were treated with one hormone and two different microorganisms in four different ways (polystimulin, biohumus, EM and EM+biohumus) and we have investigated whether these treatments have any statistically meaningful effects on corms and stigmas. It has been determined that EM + biohumus was the most effective choice for saffron cultivation, although the experimental design used did not allow us to discount the effects of other possible variables, like the presence of specific micronutrients and/or soil or aspect differences between the various plots. Moreover, additional chemical tests are required to determine the saffron quality. The positive or negative human health effects of hormone treatment of saffron, used for treating many illnesses, should also be investigated in another study. 

## Experimental

### General

**Figure 2:**
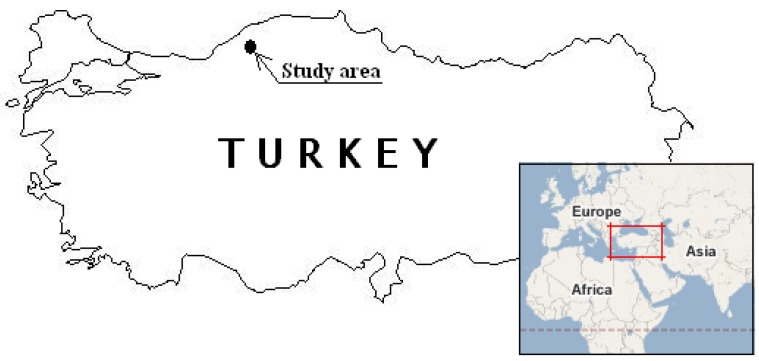
Geographical location of the study area.

The biohumus, EM, and polystimulin mixture were obtained from the Agrochemistry Research Institute of the Russian Academy of Agricultural Science. The biohumus and EM solutions were prepared adding biohumus (1 mL) or EM (1 mL) to distilled water (100 mL). The EM+biohumus solution was prepared adding EM (1 mL) and biohumus (1 mL) to distilled water (100 mL). The polystimulins, PS-A6 and PS-K, were used to break dormancy. Fifty milligrams each of PS-A6 and PS-K were prepared separately and then mixed and dissolved in alcohol (5-6 drops) and distilled water (150 mL) was added. Test plots for the study were prepared in Davutobasi village in Safranbolu, Karabuk, Turkey (Figure 2). This region has the appropriate climate and soil conditions to cultivate saffron and significant saffron cultivation has taken place in this region for many years. 

**Figure 3:**
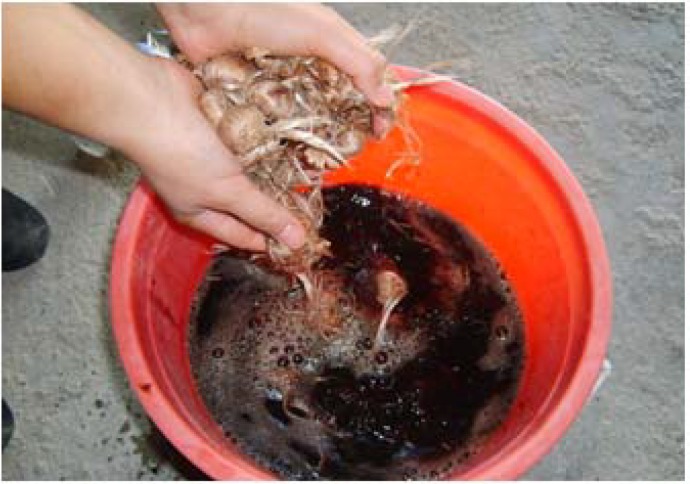
Biohumus treatment of saffron corms.

Saffron bulbs were exposed to different processes (Effective Microorganism™, Polystimulin, Biohumus and a mixture of Effective Microorganism™ and Biohumus) to examine the effect on saffron fertility and productivity. Figure 3 shows one of these treatments. Three thousand saffron bulbs were selected for each experiment. The bulbs were first visually examined and unsuitable, decayed, diseased and small-diameter (<1.5 cm) bulbs were discarded. Afterwards, these bulbs have been divided into five groups, each containing 600 bulbs. The saffron bulbs to be planted were then kept in a cool environment having no direct sunlight until planting time. Two days before planting each group of saffron bulbs was treated separately with the appropriate hormone and microorganisms and they were then incubated for 2 days. In addition, 600 control bulbs with no applied treatments were also incubated. 

### Soil Preparation

**Figure 4:**
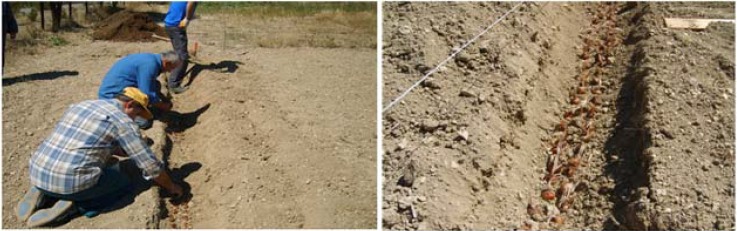
Cultivation of saffron bulbs.

The soil in which saffron is to be cultivated must be prepared very well. The farm in the test area has been fallow for a year and then it has been plowed 3 or 4 times after cleaning out weeds. Planting depth and the row spacing were set at 20-25 cm. The bulbs were not planted with regular spacing but instead placed randomly (Figure 4). The number of the bulbs planted has been between 150 and 200. 

### Fertilization

**Figure 5:**
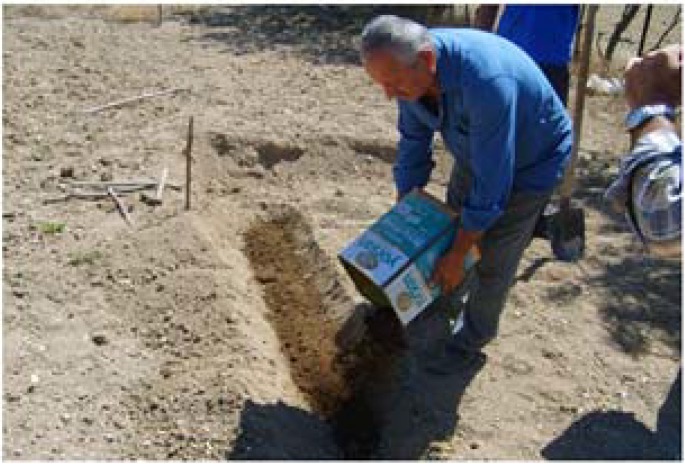
Saffron bulbs being covered with organic matter.

Planting lines were covered with soil after the bulbs were covered with matured farm fertilizer (Figure 5). There was no additional application of fertilizer.

### Bulb Harvest

The bulbs sprouted and blossomed for three years after transplanting them to a fresh plot. The bulbs were uprooted at the middle of June in the fourth year. The bulbs were transported to a warehouse after keeping them exposed to sunlight for one day to remove the moisture. 

**Figure 6:**
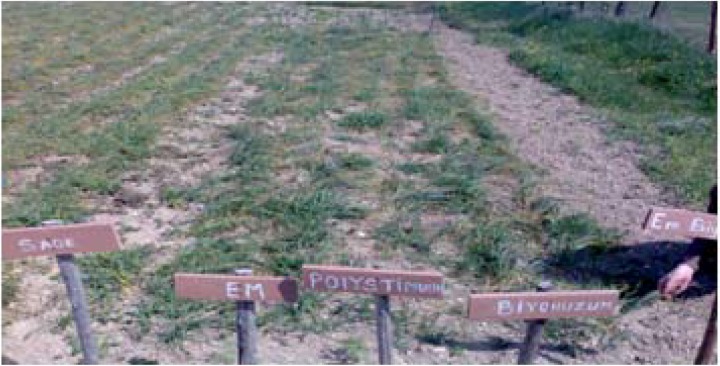
View of the test plot.

**Figure 7:**
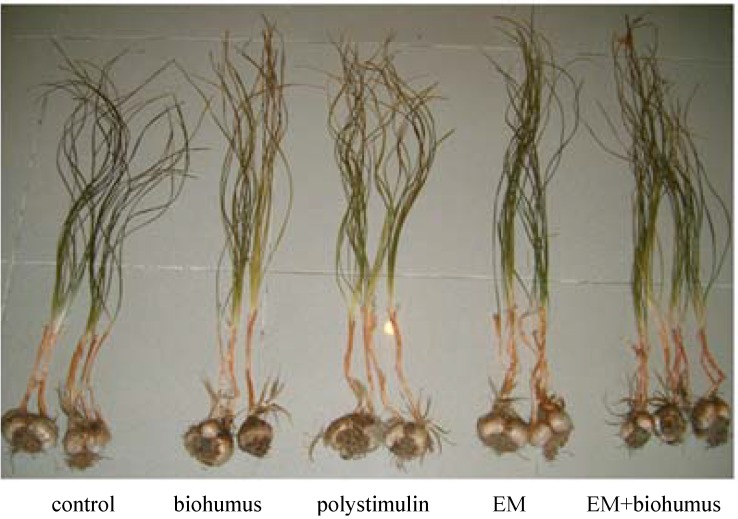
View of treated and control saffron bulbs.

The bulbs were kept in storage for four to five weeks and further prepared starting on August 20^th^ and then replanted in another plot (Figure 6). No saffron had been planted for five years on this land. At harvest time, bulbs were randomly selected and uprooted and measurements taken. Treated bulbs and tassels are shown in Figure 7. 

### Bloom and Bloom Harvest

**Figure 8:**
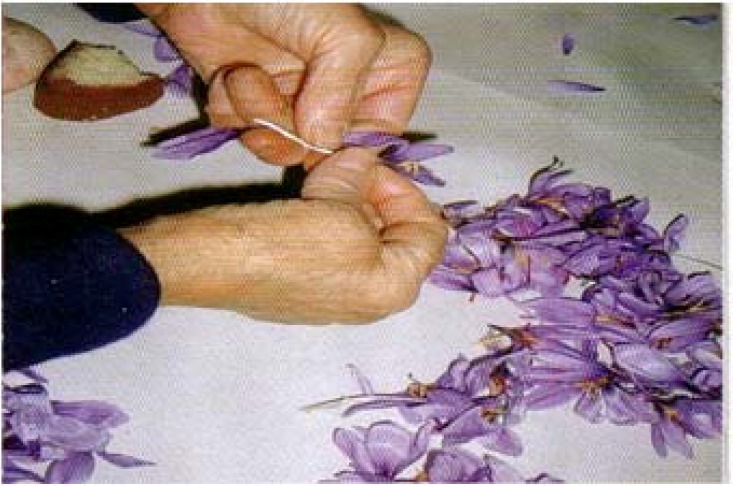
Separation of stigma and blooms.

Blooms were harvested on November 1^st^. They were gathered by hand in the early morning when the petals were not yet open. Gathered blooms were transported to a closed area and petals then opened and the stigmas and male organs were collected together (Figure 8). 

### Drying of the Stigmas

Wet stigmas were exposed to conventional drying after the measurements. Wax was poured onto the trays where the stigmas were to be dried to form a thin film in order to prevent the product from slipping and then male and female organs were loaded onto these trays. The trays are inclined against a fire and drying takes place. Measurements were performed again on saffron after the drying process was complete. Three hundred stigmas randomly selected from each group were weighed and the values obtained have been noted. 
